# A review on shared genetic architecture of endometriosis and migraine: from pleiotropy to convergent inflammatory pathways

**DOI:** 10.3389/fneur.2026.1781023

**Published:** 2026-05-15

**Authors:** Qian Yang, Shengyuan Yu

**Affiliations:** 1 Department of Neurology, Tianjin Huanhu Hospital, Tianjin, China; 2 Tianjin Key Laboratory of Cerebral Vascular and Neurodegenerative Diseases, Tianjin, China; 3 Department of Neurology, Chinese PLA General Hospital, Beijing, China

**Keywords:** endometriosis, gene, inflammatory pathways, migraine, molecular genetics

## Abstract

The comorbidity between endometriosis and migraine has long been recognized clinically, yet a unifying pathophysiological explanation has remained elusive. Traditional models, centered on hormonal fluctuations or secondary inflammation are lacking to explain the fundamental predisposition underlying their co-occurrence. This review synthesizes the evidence from genetic epidemiology that is reshaping this narrative, positing that shared molecular genetic mechanisms provide the missing link. This review paper aims to present a review of the current literature surrounding genetic overlap between EM and migraine. Critically, Mendelian Randomization analyses refute a causal relationship, instead pointing to pleiotropy as the core principle. We delve into the specific shared risk loci, such as *TRIM32* and *SLC44A4*, and demonstrate how they converge on dysregulated biological pathways, notably IL-1, TNF-*α*, and MAPK/ERK signaling that drive both peripheral inflammation in endometriosis and neuroinflammation in migraine. Central sensitization emerges as a critical amplifier, functionally coupling the two conditions and exacerbating chronic pain. By integrating these findings, we propose a novel model where endometriosis and migraine are parallel manifestations of a shared genetic architecture. Finally, we discuss the translational implications of this paradigm, including the potential for genetic stratification of high-risk patients and the repurposing of therapeutics targeting these shared inflammatory pathways. This genetic reframing may move the field beyond symptomatic management toward a future of mechanism-based, personalized medicine for this underserved patient population.

## 1 Introduction

A clinical observation has long perplexed both neurologists and gynecologists: a patient with severe, chronic migraine is significantly more likely to suffer from endometriosis, and vice versa (1, 2). This is not a rare coincidence but a robust epidemiological reality. Large-scale cohort studies and meta-analyses demonstrate that individuals with endometriosis have up to double the risk of experiencing migraine, with this comorbidity exacerbating pain, disability, and reduction in quality of life (2). This striking overlap suggests that the co-occurrence of these two conditions is not random but points to a shared biological underpinning that transcends anatomical boundaries (3, 4). The comorbidity between endometriosis and migraine can also be viewed within the broader framework of Chronic Overlapping Pain Conditions (COPCs), a group of disorders characterized by shared features of chronic pain, central sensitization, and frequent co-occurrence. For instance, studies on COPCs have demonstrated overlapping heritability between endometriosis and other pain conditions such as chronic pelvic pain and interstitial cystitis, supporting the concept of common biological susceptibilities underlying these disorders (5).

Traditionally, the pathophysiological understanding of these disorders has been siloed within separate medical disciplines. Endometriosis is predominantly explained by Sampson’s theory of retrograde menstruation and the subsequent establishment of inflammatory lesions within the pelvic cavity (6, 7). Conversely, migraine is understood through the neurological lens of the trigeminovascular system, involving cortical spreading depression and the release of vasoactive neuropeptides. While these models are valuable, they are inherently limited, offering no clear explanation for why the same individual would be susceptible to both (8). Central to this is cortical spreading depression (CSD), a self-propagating wave of neuronal and glial depolarization across the cerebral cortex, followed by a prolonged suppression of neural activity. CSD underlies the migraine aura and activates the trigeminovascular system, resulting in the release of vasoactive neuropeptides and neurogenic inflammation (9). Importantly, this phenomenon illustrates how neuronal excitability and neuroinflammatory processes are intertwined, providing a potential mechanistic bridge to systemic inflammatory conditions such as endometriosis (10). Numerous hypotheses, such as the role of fluctuating estrogen or systemic inflammation, provide a correlative bridge but fail to identify the fundamental, predisposing factors that unite these conditions at their root (4, 11, 12).

**Figure 1:**
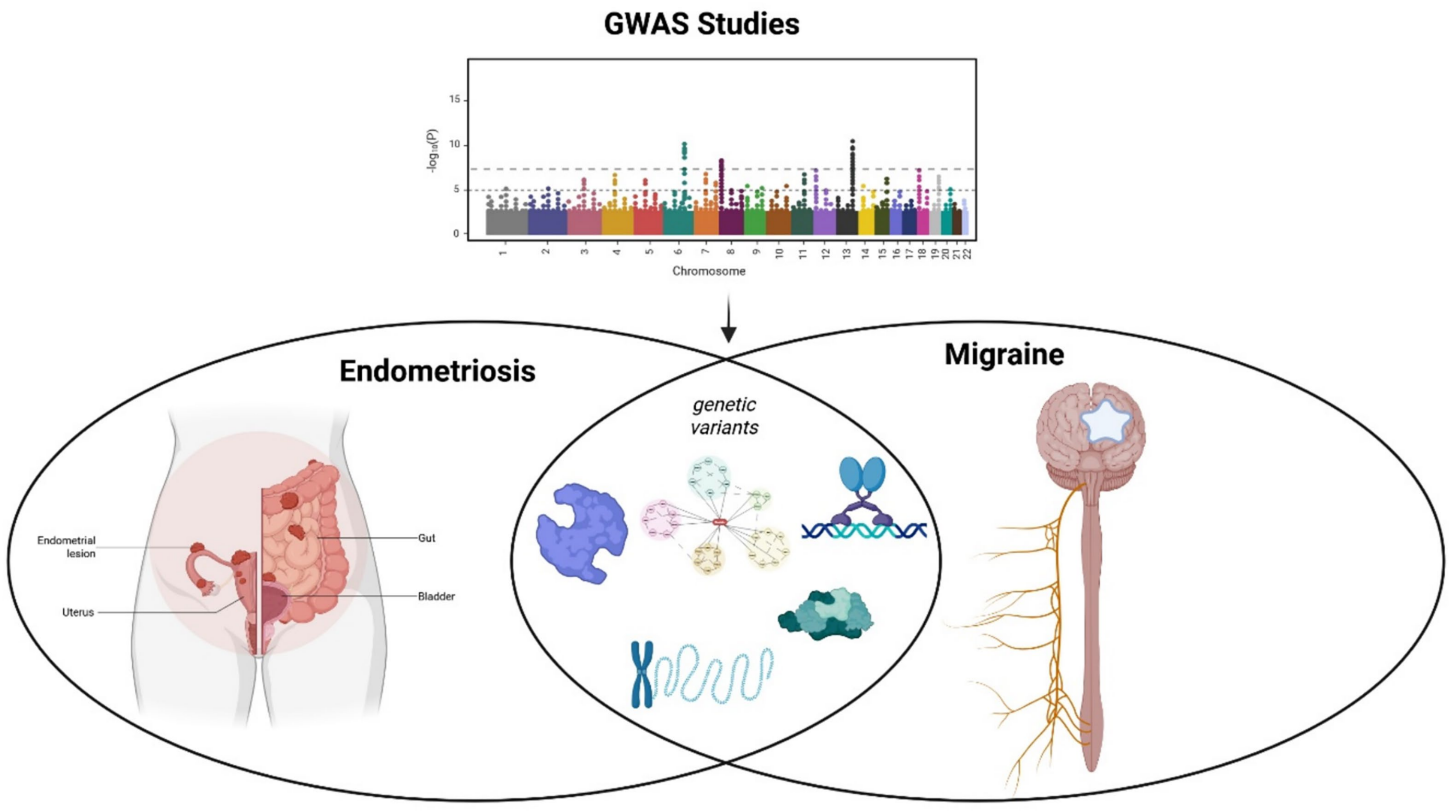
Genome-wide association studies reveal a shared genetic architecture underlying the migraine-endometriosis comorbidity. Genome-wide association studies (GWAS) have addressed this gap by demonstrating genetic pleiotropy, identifying the specific genes and pathways that constitute a shared biological substrate predisposing individuals to both conditions. Created with Biorender.

This gap in understanding leads to a pivotal question: what is the intrinsic biological link that predisposes an individual to develop both endometriosis and migraine? Recognizing endometriosis and migraine as components of the COPC spectrum provides a conceptual framework that integrates genetic pleiotropy, neuroimmune dysregulation, and central sensitization as shared drivers of disease risk (5). If hormonal fluctuations or inflammation alone were sufficient, the comorbidity would be nearly universal, which it is not. There must be a foundational element that primes certain physiological systems to respond abnormally to these triggers, leading to the manifestation of both disorders. The answer is emerging from the field of genetics. Recent advances in genomic science provide a unifying, mechanistic explanation for this comorbidity through the principle of pleiotropy; whereby, a single genetic variant influences multiple, seemingly distinct traits. Groundbreaking genome-wide association studies (GWAS) have moved beyond simply confirming the heritability of each disorder individually; they have begun to map the specific shared genetic architecture that underlies their co-occurrence (Figure 1) (4, 11). This represents a paradigm shift, suggesting that endometriosis and migraine are not merely associated but are different clinical expressions of a common genetic substrate.

Therefore, the aim of this review is to synthesize this transformative body of evidence. We will first delineate the epidemiological landscape that frames the clinical problem. Next, we will then examine the genetic data revealing significant correlation and shared risk loci. Finally, we will explore how these shared genetic variants converge on key biological pathways, such as inflammation and pain sensitization to create a unified pathophysiological model. We will conclude the profound translational implications of this genetic paradigm for redefining patient subgroups, repurposing existing therapies, and pioneering novel, targeted interventions for this underserved patient population.

## 2 The epidemiological landscape

### 2.1 The individual burden of disease

Endometriosis and migraine are two prevalent chronic pain disorders affecting women globally, each imposing a formidable individual burden. Endometriosis, characterized by the presence of endometrial-like tissue outside the uterine cavity, is a complex gynecological condition affecting an estimated 1 in 10 women of reproductive age (13–15). Its pathology is driven by chronic inflammation, leading to a group of symptoms that extend beyond the classic triad of severe dysmenorrhea, deep dyspareunia, and infertility. Patients often endure chronic pelvic pain, dyschezia, and profound fatigue, resulting in significant impairments to physical, mental, and social well-being. The diagnostic delay, often spanning 7–10 years, exacerbates this burden, allowing disease progression and compounding patient suffering (16–19).

Similarly, migraine is not merely a bad headache but a hereditary, complex neurological disorder. It is marked by recurrent, often unilateral, pulsating headaches of moderate to severe intensity, typically lasting 4 to 72 h and accompanied by photophobia, phonophobia, and nausea (20). With a global prevalence of approximately 14% and a 3:1 female-to-male preponderance, migraine is a leading cause of years lived with disability worldwide. The disorder is characterized by altered sensory processing and, for a significant subset, the disabling neurological phenomena of aura. The unpredictable nature of attacks creates a state of constant apprehension, disrupting education, employment, and daily activities, and placing an economic burden on healthcare systems and society (21–23).

### 2.2 The compounding burden of comorbidity

Previous studies reported that when these two challenging conditions converge in the same individual, the burden is not simply additive; it is synergistic, creating a compounded clinical picture far more severe than the sum of its parts (3, 24). A previous study demonstrate a significantly increased risk of migrainous headache in women with endometriosis compared to women without endometriosis (odds ratio [OR] 1.57, 95% confidence interval [CI]: 1.12–2.21, *p* = 0.009) (25). This risk appears to be dose-dependent, with studies indicating that the prevalence and severity of migraine increase with the anatomical stage of endometriosis. For instance, women with advanced-stage (Stage III/IV) disease exhibit an even higher likelihood of co-morbid migraine, suggesting a shared underlying pathophysiology that escalates in tandem with disease severity (26).

The clinical repercussions of this comorbidity are profound. Patients suffering from both conditions report higher pain scores, pain chronicity, and an advance state of central sensitization compared to those with either condition alone (3, 27). This translates into a severely diminished quality of life, higher rates of anxiety and depression, and functional impairment. From a healthcare perspective, these individuals are high utilizers of medical services, navigating multiple specialties and undergoing numerous diagnostic tests and treatments, often with suboptimal outcomes due to the siloed nature of their care (28). This clinical overlap, observed across diverse populations and study designs, cannot be dismissed as random chance. It serves as a clue that points to an intrinsic biological link, a shared vulnerability that predisposes a subset of women to develop both disorders, setting the stage for a genetic investigation (12, 28).

## 3 The genomic revolution: unveiling shared heritability

The epidemiological link between endometriosis and migraine demanded an explanation that moved beyond observable symptoms and into the fundamental blueprint of biology: the genome. The advent of large-scale genetic studies has provided the tools to do just this, shifting the investigation from clinical correlation to molecular mechanism. By analyzing the DNA of hundreds of thousands of individuals, researchers have begun to unravel the shared heritability that underlies this comorbidity, revealing a relationship that is more fundamental (4, 11).

### 3.1 Decoding the genome’s secrets

Three key methodological advances are critical in this pursuit. First, GWAS function as a discovery engine. By scanning the genomes of vast cohorts, GWAS identifies single-nucleotide polymorphisms (SNPs), common genetic variants that occur frequently in individuals with a specific disease compared to healthy controls (29, 30). Each identified SNP is a signpost pointing to a genomic region involved in the disease’s biology. Second, LD Score Regression (LDSC) builds upon GWAS data to answer a critical question: to what extent do the genetic influences on two different traits overlap? It calculates a genetic correlation (rG), a statistic ranging from −1 to 1 (31). A positive genetic correlation indicates that shared genetic variants contribute to both endometriosis and migraine, suggesting overlapping genetic architecture rather than direct causality. Mendelian randomization (MR) analyses using available instruments generally do not support a strong causal effect in either direction, highlighting instead a shared genetic basis (4). However, MR findings should be interpreted cautiously, as they depend on assumptions such as instrument strength, absence of pleiotropy, and consistent application of bidirectional analyses (32). The GWAS and LDSC studies cited in this review report modest but significant positive genetic correlations between endometriosis and migraine, supporting the presence of shared heritable risk rather than direct causal effects.

### 3.2 Establishing the genetic link

The application of this genomic toolbox has reshaped our understanding of the endometriosis-migraine relationship. It has long been established that both disorders are individually heritable. Family and twin studies (25, 33) consistently show that both endometriosis and migraine cluster in families, implying a genetic component to their etiology. However, this alone did not explain their co-occurrence (3, 4).

The advancement came from studies applying LD Score Regression to large GWAS datasets. These analyses revealed a significant positive genetic correlation (rG) between endometriosis and migraine (25). A seminal 2021 meta-analysis, for instance, found a genetic correlation of approximately 0.38, a substantial overlap that provides the first statistical proof of a shared genetic basis (4). This finding means that a non-trivial portion of the genetic variants that elevate a woman’s risk for endometriosis also, independently, elevate her risk for migraine. It is the molecular explanation for the clinical observation, the same underlying genetic architecture predisposes an individual to both conditions (25).

Perhaps the notable insight comes from Mendelian Randomization analyses. When researchers used genetic instruments for endometriosis to test for a causal effect on migraine (and vice-versa), the results were clear: there was no evidence of a causal relationship (4). It refutes the simplistic model that chronic pain from endometriosis directly “causes” migraine, or that migraine attacks somehow predispose to the development of endometriotic lesions. Instead, the evidence strongly points to pleiotropy as the underlying mechanism (4, 11). The comorbidity is not a chain of cause and effect but rather the parallel manifestation of a shared genetic predisposition. It is the same root a common set of genetic variants affecting key biological pathways, giving rise to two different branches of disease. This reframing moves the scientific question from “Which one causes the other?” to the more profound and actionable: “What are these shared genetic pathways, and how do they mechanistically lead to both disorders?”

## 4 From correlation to mechanism: key shared genes and pathways

The findings of a genetic correlation between endometriosis and migraine was a crucial first step, but it was akin to knowing two cities are connected by a busy highway without knowing what goods are being transported (25). The subsequent challenge is to identify the specific genetic “vehicles” (shared risk loci) and, more importantly, the common biological “cargo” (pathways) they carry that ultimately drive both diseases. This transition from statistical correlation to mechanistic understanding is where the true explanatory power of genetics lies, revealing a pathophysiological hub where these two disorders converge.

### 4.1 Pinpointing the players: shared risk loci

**Figure 2:**
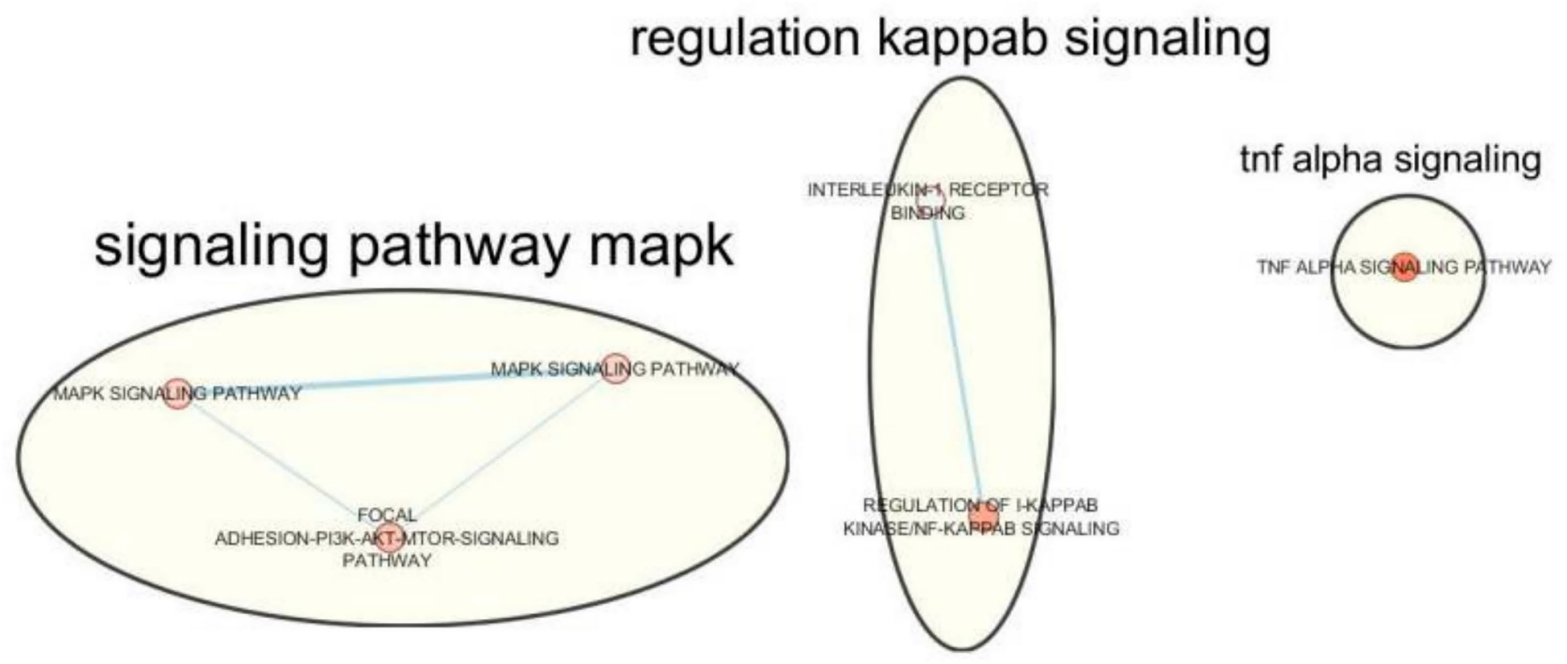
Clustered biological themes for overlapping endometriosis migraine genes. Reproduced with permission from Adewuyi and Sapkota (4).

A previous study demonstrates that endometriosis and migraine co-occur due to shared, non-causal genetic mechanisms, identifies novel pleiotropic risk genes and inflammatory signaling pathways, and highlights the need for integrated clinical screening and future functional validation (4). TRIM32 (Tripartite Motif Containing 32) encodes an E3 ubiquitin ligase involved in protein turnover and cellular homeostasis (34, 35) and is expressed in neural tissues with roles in neurodevelopment. Its identification as a shared locus suggests that altered ubiquitin-mediated regulation may contribute to disease comorbidity. Dysregulation of TRIM32 could plausibly affect inflammatory signaling in pelvic tissues and neuronal excitability within pain pathways, thereby increasing susceptibility to both endometriosis progression and migraine attacks (4). SLC44A1 (Solute Carrier Family 44 Member 1) belongs to a choline transporter family critical for acetylcholine synthesis, membrane signaling, and inflammatory regulation (36). Given the established anti-inflammatory and neuromodulatory roles of cholinergic signaling (37), impaired SLC44A1 function may represent a shared mechanism linking dysregulated peritoneal inflammation with altered central pain modulation relevant to migraine (38). Figure 2 illustrate the biological themes for overlapping endometriosis–migraine genes.

Although cross-trait GWAS analyses support TRIM32 and SLC44A1 as shared risk loci, current evidence primarily establishes genetic association rather than definitive mechanistic causality. Published studies indicate that TRIM32 participates in ubiquitin-mediated protein regulation and neural development (39), and that SLC44A1 contributes to choline transport and cholinergic signaling, processes broadly implicated in inflammation and neuromodulation (36). However, their proposed roles in linking endometriosis and migraine through shared inflammatory or pain-processing pathways should be considered hypothesis-driven and require targeted functional validation in disease-relevant tissues. Beyond these genes, additional loci related to sex steroid signaling and neuro-inflammatory pathways are repeatedly suggested across genetic studies, even when not meeting stringent cross-trait significance thresholds. Collectively, these findings point to biologically coherent, functionally plausible candidates that provide a focused framework for downstream experimental validation (11).

### 4.2 The convergent pathways: a pathophysiological hub

While individual genes provide important clues, pathway-level analyses reveal that the shared genetic architecture of endometriosis and migraine converges on a small number of interconnected inflammatory networks, forming a core pathophysiological hub (4).

The enriched pathways involve pro-inflammatory signaling, particularly Interleukin-1 (IL-1), Tumor Necrosis Factor-*α* (TNF-α), and the MAPK/ERK cascade (Figure 2) (40, 41).

IL-1 and TNF-α act as upstream regulators of inflammatory responses in both conditions. In endometriosis, elevated levels in peritoneal fluid promote lesion growth, angiogenesis, and pain sensitization (42), whereas in migraine these cytokines activate the trigeminovascular system, sensitize meningeal afferents, and facilitate the release of neuropeptides such as CGRP and substance P, driving neurogenic inflammation (43–45). The shared genetic enrichment of these pathways suggests a predisposition to exaggerated inflammatory signaling that manifests in tissue-specific contexts, including the pelvis and meninges.

**Figure 3:**
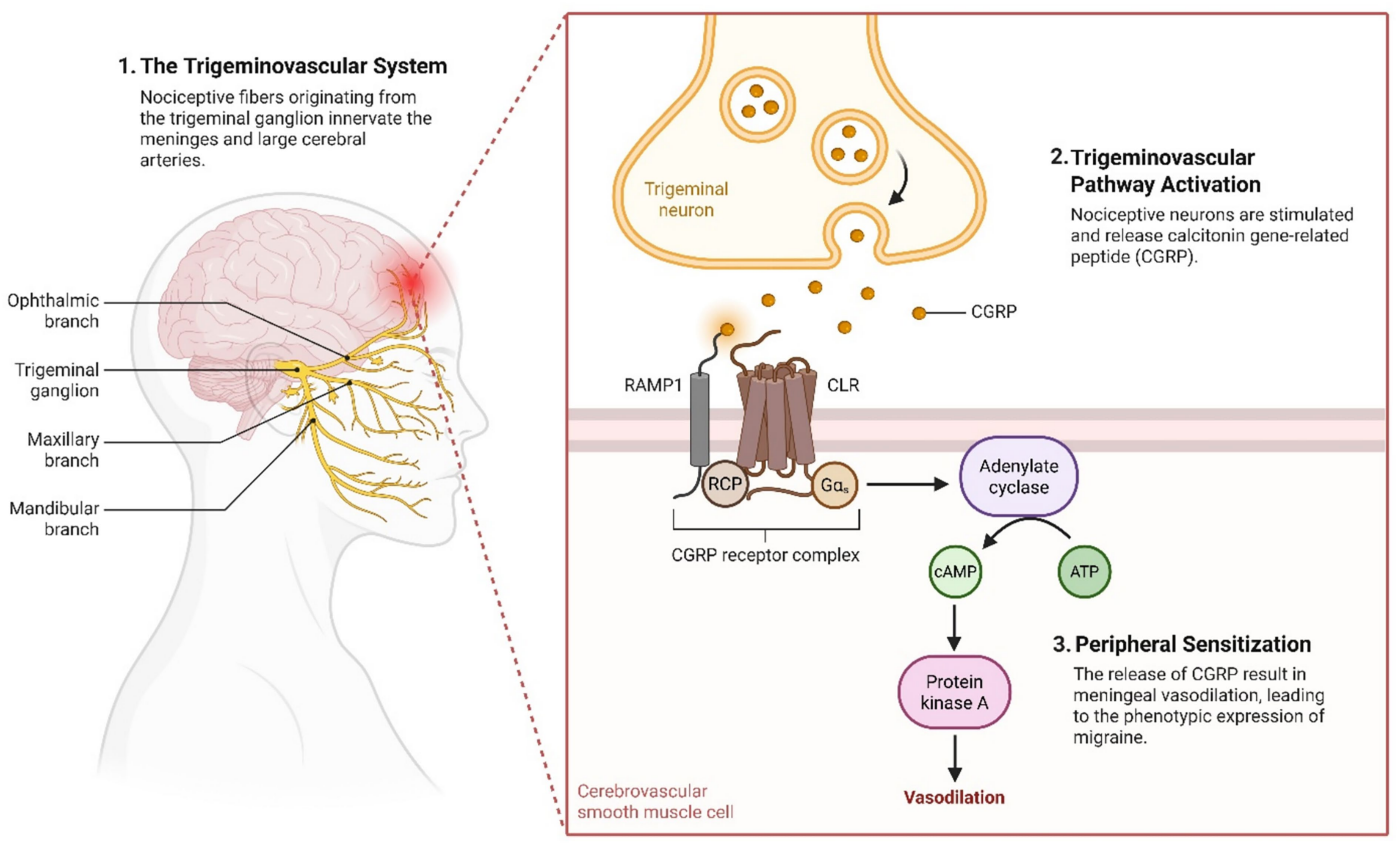
The trigeminovascular system regulates cranial blood vessels and transmits pain signals. Calcitonin gene-related peptide (CGRP), abundant in trigeminal nerve fibers, is a key mediator of migraine, with its peripheral release triggering cerebral vasodilation and the onset of migraine pain. Created with Biorender.

Consistent with this framework, calcitonin gene-related peptide (CGRP) plays a central role in migraine pathophysiology by promoting vasodilation and neuroinflammation, further linking inflammatory and neuronal mechanisms within this shared biological axis (Figure 3) (46).

**Figure 4:**
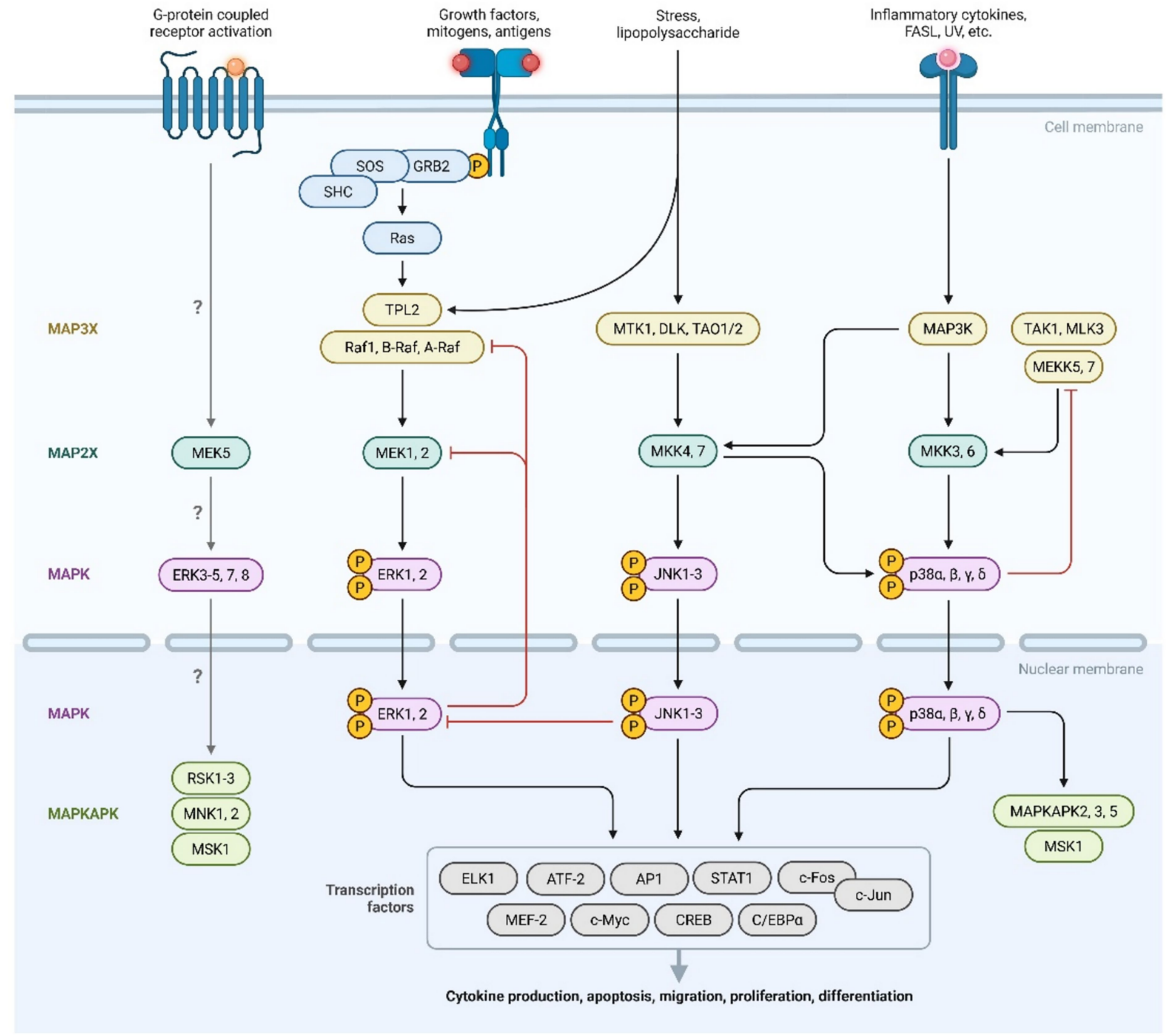
MAPK signaling pathway. Created with Biorender.

The MAPK/ERK pathway is a central intracellular signaling cascade that regulates cell proliferation, survival, and inflammatory responses (47). In endometriosis, MAPK/ERK activation supports the survival and invasive capacity of ectopic endometrial cells, whereas in migraine it has been implicated in cortical spreading depression and pain sensitization within the trigeminal system (48). Genetic variants that enhance MAPK/ERK signaling may therefore exert pleiotropic effects by promoting lesion persistence in the pelvis while lowering the threshold for neuronal excitability in migraine-relevant circuits (Figure 4) (4). Genome-wide association studies identifying shared risk loci between endometriosis and migraine, including genes such as *TRIM32*, form the basis for downstream pathway enrichment analyses. These analyses reveal a significant convergence on immune and stress-response signaling cascades, particularly the MAPK/ERK, IL-1, and TNF-*α* pathways. Thus, the pathway-level findings represent a functional interpretation of shared genetic susceptibility rather than an independent line of evidence (4).

## 5 Pain and sensitization

This shared inflammatory signaling environment may contribute to central sensitization, a state of heightened responsiveness within the central nervous system. Persistent nociceptive input from inflamed endometriotic lesions can facilitate synaptic plasticity in spinal and brainstem pain pathways, a process further amplified by pro-inflammatory cytokines such as IL-1β, which act as neuromodulators (43, 49). Central sensitization is a recognized contributor to migraine chronification, providing a mechanistic link through which peripheral inflammatory pathology and central pain processing may interact (50).

Within this framework, estrogen is best viewed as a modulator rather than a primary driver of disease. Estrogen signaling influences multiple inflammatory and sensory pathways, including MAPK/ERK, and may amplify genetically determined inflammatory and pain-sensitization processes (51, 52). This interaction offers a plausible explanation for the cyclical nature of symptoms in both conditions and for the variable efficacy of hormonal therapies, which modulate pathway activity without addressing the underlying genetic susceptibility (53, 54). Substance P (SP) has been recognized as a key mediator in migraine through neurogenic inflammation and pain sensitization (55). Emerging evidence also implicates SP in endometriosis, where elevated levels and increased NK1R expression in lesions contribute to hyperalgesia and local neuroinflammation. These findings suggest a shared role of SP in pain mechanisms across both conditions.

Central sensitization is increasingly recognized as a core biological mechanism underlying Chronic Overlapping Pain Conditions, including both endometriosis and migraine. Contemporary models emphasize the role of neuroinflammatory processes in sustaining heightened central nervous system excitability, whereby persistent peripheral nociceptive input promotes microglial and astrocytic activation, cytokine release, and maladaptive synaptic plasticity (56). Pro-inflammatory mediators such as IL-1β and TNF-*α* amplify excitatory neurotransmission and suppress inhibitory signaling within pain-processing circuits, resulting in exaggerated and persistent pain responses. Importantly, emerging genetic evidence suggests that inherited variation in immune and neuroinflammatory pathways may predispose individuals to central sensitization (57–59), providing a mechanistic link between shared genetic architecture and multisite chronic pain phenotypes.

Together, these findings support a model in which endometriosis and migraine arise from shared genetic influences converging on inflammatory and sensory processing pathways. This perspective reframes their comorbidity as the result of overlapping biological mechanisms rather than coincidental coexistence and highlights common molecular targets for future mechanistic and therapeutic studies (Table 1). These findings position central sensitization not merely as a downstream consequence, but as a genetically primed process that functionally links shared inflammatory signaling to chronic pain across endometriosis and migraine.

**Table 1:** Convergent biological pathways in endometriosis and migraine pathogenesis.

Pathway	Role in endometriosis	Role in migraine	Shared genetic and molecular drivers
IL-1/TNF-α Signaling	Drives proliferation of lesions, angiogenesis, and production of pain-sensitizing prostaglandins in the peritoneal environment.	Activates the trigeminovascular system, sensitizes meningeal afferents, and promotes release of CGRP, leading to neurogenic inflammation.	Enrichment of shared variants in genes regulating the NF-κB cascade.
MAPK/ERK Signaling	Promotes survival, proliferation, and invasion of ectopic endometrial cells.	Mediates cortical spreading depression and pain sensitization within the trigeminal nucleus caudalis.	Genetic correlation implicating genes in the Ras/Raf/MEK/ERK cascade.
Central Sensitization	Persistent nociceptive input from pelvic lesions causes wind-up in the spinal cord and brainstem.	Lowers the threshold for migraine attacks and contributes to chronification of headache.	Shared genetic predisposition to a hyperexcitable CNS and heightened inflammatory tone.

## 6 Synthesis: a proposed integrated model

The accumulated genetic and pathophysiological evidence necessitates a paradigm shift from viewing endometriosis and migraine as merely associated conditions to understanding them as different clinical manifestations of a shared biological substrate (60–62). To crystallize this concept, we propose an integrated model that moves from a common genetic root to divergent disease phenotypes, unified by a central amplifier of pain.

### 6.1 The foundation: a shared genetic susceptibility

At the base of the model lies the shared genetic architecture, composed of specific pleiotropic risk loci such as *TRIM32* and *SLC44A1* (4), and a broader background of genetic variants identified through their positive correlation. This genetic foundation does not code for either disease specifically but rather for a predisposed physiological state (63). It represents the inherent, inherited vulnerability that sets the stage for the entire cascade. This layer answers the “why” question—why certain individuals are susceptible to both conditions while others are not. It is the fundamental, non-negotiable element that must be present for this specific comorbidity to manifest (64).

### 6.2 The mechanism: core pathway dysregulation

The shared genetic variants do not remain silent; they exert their influence by biasing key biological systems toward a state of hyper-reactivity. This is the mechanistic core of the model, where genetic risk translates into functional dysregulation. The primary pathways involved are the pro-inflammatory signaling cascades (specifically IL-1 and TNF-*α*) and the MAPK/ERK growth/signaling pathway (65–68). The genetic predisposition lowered threshold for the activation of these pathways and an exaggerated response when they are triggered. This dysregulation creates a system-wide propensity for heightened inflammation and cellular excitability. Environmental factors, such as hormonal fluctuations, surgical injury, or stress, act upon this primed system, serving as triggers that ignite these dysregulated pathways (69). The dysregulated core pathways, once activated, manifest clinically in a tissue-specific manner, leading to the distinct diagnoses of endometriosis and migraine.

In pelvic and reproductive tissues, the hyperactive inflammatory and MAPK pathways drive the hallmarks of endometriosis: proliferation of ectopic endometrial cells, angiogenesis, and the secretion of proanalgesic (pain-producing) mediators that create a state of persistent peripheral nociception (70, 71).

In the trigeminovascular system and central nervous system (CNS), the same inflammatory milieu (e.g., IL-1β, TNF-*α*) directly sensitizes meningeal afferents and lowers the activation threshold of the trigeminal nerve. The dysregulated MAPK signaling contributes to cortical spreading depression and central pain facilitation. This results in the episodic and chronic pain that characterizes migraine (72, 73).

### 6.3 The amplifier: a vicious cycle of central sensitization

The model does not end with two parallel conditions. It incorporates a feedback loop that acts as a central amplifier: central sensitization (58). The persistent nociceptive input from the pelvic cavity in endometriosis continuously bombards the dorsal horn of the spinal cord and ascending pain pathways, leading to a wind-up phenomenon and a state of hyperexcitability in the CNS. This globally sensitized state directly feeds into and exacerbates the migraine phenotype (74, 75), making attacks frequent and severe. Conversely, the recurrent trigeminal activation of migraine may further tune this sensitized circuit. Central sensitization thus becomes the “final common pathway” for chronic pain, functionally coupling the two conditions and creating a self-reinforcing vicious cycle that explains the compounded clinical burden observed in comorbid patients.

In conclusion, this integrated model provides a framework that links the statistical finding of genetic correlation to the clinical reality of comorbid suffering. It posits that endometriosis and migraine are not one causing the other, but parallel outcomes of a shared genetic predisposition that dysregulates core inflammatory and signaling pathways, whose manifestations are powerfully linked and amplified through the central nervous system. This reframing offers a new lens for research and therapeutic development, suggesting that interventions targeting these shared foundational mechanisms could yield benefits for both conditions simultaneously.

## 7 Future directions and translational implications

The identification of a shared genetic architecture between endometriosis and migraine provides a framework for translating genetic discovery into functional insight and clinical application. Progress in this field now requires a shift from association-based analyses toward mechanistic validation and therapeutic development that explicitly acknowledge shared inflammatory and sensory pathways.

### 7.1 From genetics to function: validating the shared biology

Pleiotropic loci such as *TRIM32* and *SLC44A4*, together with pathway-level enrichment of inflammatory signaling, represent important starting points but require functional validation to establish causal relevance (76). Moving from statistical association to biological mechanism will necessitate complementary *in vitro* and *in vivo* approaches (77, 78). For example, co-culture systems incorporating dorsal root ganglion neurons and endometriotic stromal cells from matched genetic backgrounds could be used to assess how manipulation of shared genes influences neuronal excitability and inflammatory mediator release (79, 80). In parallel, animal models with targeted modulation of pathways such as MAPK/ERK could be evaluated for pain-related behaviors relevant to both visceral and trigeminal sensitization (81). While genetic and pathway analyses support the involvement of loci such as *TRIM32* and *SLC44A4*, their proposed roles in linking inflammatory signaling and neuronal hyperexcitability remain hypothesis-driven and require tissue-specific experimental confirmation.

### 7.2 Redefining patient subgroups: the dawn of genetic stratification

The shared genetic basis of endometriosis and migraine offers an opportunity to move beyond symptom-based classification toward biologically informed patient stratification. Development of polygenic risk scores for comorbidity could help identify individuals at elevated risk for developing chronic or overlapping disease phenotypes (82). Such stratification may inform earlier intervention strategies aimed at mitigating central sensitization and could improve clinical trial design by enriching study populations for patients most likely to benefit from therapies targeting shared mechanisms. This approach supports a shift from reactive symptom management toward proactive prevention of comorbidity-associated disease burden.

### 7.3 Drug repurposing and novel therapeutics: targeting the root cause

Pathway convergence provides a rationale for evaluating existing therapies for cross-condition benefit. Agents targeting upstream inflammatory mediators, such as IL-1 or TNF-*α*, may offer therapeutic potential in genetically stratified patients with comorbid endometriosis and migraine, although safety and tolerability considerations necessitate careful clinical evaluation (83–85). Similarly, modulators of MAPK/ERK signaling, currently explored in other disease contexts, warrant investigation for their capacity to influence both lesion-associated inflammation and pain sensitization (86). Targeting shared upstream mechanisms contrasts with condition-specific approaches, such as CGRP inhibition in migraine, which may alleviate headache symptoms without addressing pelvic pain. A network-based therapeutic strategy focused on common biological pathways may therefore offer broader clinical benefit for patients with overlapping disease manifestations.

Notably, a pleiotropic genetic framework also suggests that some widely used treatments may already exert cross-disciplinary effects. For example, amitriptyline is prescribed for both migraine prevention and chronic pelvic pain, potentially reflecting modulation of shared neuroinflammatory or pain-sensitization pathways. Likewise, non-steroidal anti-inflammatory drugs and dietary anti-inflammatory interventions may influence common biological processes underlying comorbid disease, supporting a more integrated, mechanism-based approach to treatment.

Together, these findings position shared genetics as a foundation for mechanistic discovery, patient stratification, and therapeutic innovation. By validating biological mechanisms, refining risk-based patient classification, and targeting shared molecular pathways, future research can move toward more effective, integrated management strategies for individuals affected by both endometriosis and migraine (Table 2).

**Table 2:** Translational opportunities: from shared genetics to novel therapeutics.

Therapeutic strategy	Candidate agents	Testable hypothesis for clinical trial	Challenges and considerations
IL-1 inhibition	Anakinra, Rilonacept	In women with comorbid endometriosis and migraine, an IL-1 antagonist will reduce both chronic pelvic pain scores and monthly migraine days.	Systemic side effects; requires subcutaneous injection.
TNF-α antagonism	Adalimumab, Etanercept	TNF-α inhibition will improve pain-specific quality of life in a refractory, genetically-stratified comorbid population.	Significant immunosuppressive risk; better for proof-of-concept than widespread use.
MAPK pathway modulation	Selumetinib, other MEK inhibitors	A brain-penetrant MEK inhibitor will attenuate both lesion growth in animal models and associated pain behaviors.	Oncology drugs require careful re-dosing for chronic pain; toxicity profiles.
Genetic stratification	Polygenic Risk Score (PRS)	A high comorbidity PRS will predict a superior response to therapies targeting shared pathways (e.g., IL-1 inhibitors) compared to standard care.	Requires validation in large, diverse cohorts; implementation in clinical workflow.

A shared pleiotropic genetic framework suggests that several currently available therapies may already exert cross-benefit across conditions traditionally managed in different clinical disciplines. For example, amitriptyline is commonly used for both migraine prevention and chronic pelvic pain, potentially reflecting modulation of shared neuroinflammatory or pain-sensitization pathways. Similarly, non-steroidal anti-inflammatory drugs and dietary anti-inflammatory interventions may influence common biological mechanisms underlying endometriosis-associated migraine, supporting a more integrated, mechanism based treatment approach.

### 7.4 Broader symptom clustering and multisystem comorbidity

Beyond migraine, endometriosis is accompanied by a cluster of pain and non-pain conditions, including irritable bowel syndrome, bladder pain syndrome, pelvic myalgic syndromes, chronic fatigue, and mood disorders. Prior work has shown that these comorbidities commonly co-occur in adolescents and young women with endometriosis, supporting a shared underlying vulnerability rather than independent disease entities (87). Although the present review focuses on migraine, the genetic mechanisms discussed particularly pleiotropy, inflammatory pathway dysregulation, and altered pain processing are likely relevant to these additional conditions, many of which are managed across different clinical disciplines. This broader perspective supports viewing endometriosis as a systemic disorder with multisystem manifestations and shared genetic drivers.

## 8 Conclusion

The comorbidity between endometriosis and migraine has long been a clinical puzzle, its pieces scattered across the disparate domains of gynecology and neurology. The evidence synthesized in this review consolidates these pieces into a coherent picture, revealing that this relationship is not one of chance or simple correlation, but a powerful paradigm of pleiotropy. The significant genetic correlation, the identification of shared risk loci, and the convergence on core inflammatory and sensory pathways collectively demonstrate that these disorders are different clinical manifestations of a common genetic substrate.

This understanding provides a detailed explanation; it offers a transformative, mechanistic framework that reshapes our approach. It moves the clinical observation from a statistical association to a biological fate rooted in an individual’s genome. This framework bridges the disciplinary divides that have historically fragmented patient care, creating a unified pathophysiological model where peripheral pelvic inflammation and central nervous system sensitization are seen as parallel outcomes of the same underlying dysregulation. Importantly, this genetic paradigm shifts the therapeutic silos that have limited progress. By shifting the focus from the symptoms of each disease to their shared genetic and molecular roots, it may open a new frontier for developing targeted, effective “double-duty” therapies. For the millions of patients whose lives are dominated by the dual burden of endometriosis and migraine, this unified understanding heralds a future of precision medicine, where treatment is not just about managing separate conditions, but about addressing the singular, shared biological vulnerability that connects them.
